# 

**Published:** 1888-03

**Authors:** 


### CONTENTS.

### COMMUNICATIONS.

Inflammation about the Third Molar .................................... 109
Orthodontia .................................................................. 115
Monograph of the First Permanent Molar................................ 120
Rubber in Prosthetic Dentistry............................................ 131

### SELECTIONS.

Dental Irritation as a Factor in the Causation of Epilepsy ........ 138
A Case of Hæmoptysis........................................................ 147
The Duty on Medical Supplies ............................................. 148
Naphthol as an Antiseptic .................................................. 149
Teeth in Inherited Syphilis ................................................. 150
The Influence of Heredity upon Deformities and Defects .......... 150
SALMAGUNDI.................................................................. 151

### CORRESPONDENCE.

Indiana State Board of Dental Examiners................................ 154
American Medical Association.............................................. 155

### EDITORIAL.

Progress in Anæsthesia ...................................................... 157
Michigan State Dental Society.............................................. 159
Correction ...................................................................... 159
Removal.......................................................................... 160

